# Common Features of the Pericentromere and Nucleolus

**DOI:** 10.3390/genes10121029

**Published:** 2019-12-10

**Authors:** Colleen J. Lawrimore, Kerry Bloom

**Affiliations:** Department of Biology, University of North Carolina at Chapel Hill, Chapel Hill, NC 27599-3280, USA; clonerga@email.unc.edu

**Keywords:** pericentromere, nucleolus, condensin, cohesin, rDNA

## Abstract

Both the pericentromere and the nucleolus have unique characteristics that distinguish them amongst the rest of genome. Looping of pericentromeric DNA, due to structural maintenance of chromosome (SMC) proteins condensin and cohesin, drives its ability to maintain tension during metaphase. Similar loops are formed via condensin and cohesin in nucleolar ribosomal DNA (rDNA). Condensin and cohesin are also concentrated in transfer RNA (tRNA) genes, genes which may be located within the pericentromere as well as tethered to the nucleolus. Replication fork stalling, as well as downstream consequences such as genomic recombination, are characteristic of both the pericentromere and rDNA. Furthermore, emerging evidence suggests that the pericentromere may function as a liquid–liquid phase separated domain, similar to the nucleolus. We therefore propose that the pericentromere and nucleolus, in part due to their enrichment of SMC proteins and others, contain similar domains that drive important cellular activities such as segregation, stability, and repair.

## 1. Introduction

During cell division, the centromere functions as an essential genetic locus for ensuring faithful chromosome segregation. Microtubules connect to each centromere on sister chromatids via a proteinaceous complex called the kinetochore. Eukaryotic centromeres range in complexity from simple point centromeres in budding yeast to regional centromeres in fission yeast, plants, and mammals [1,2]. In budding yeast, the pericentromere is defined as the cohesin- and condensin-enriched region spanning 30–50 kb on either side of the conserved centromeric sequence [3,4,5]. However, in regional centromeres, which are enriched in heterochromatic alpha satellite repeats, there are multiple sites of microtubule attachment. Despite these differences, the interkinetochore distance is conserved among eukaryotes [6], suggesting an important conservation of centromere mechanics.

The nucleolus, as the site of ribosome biogenesis, arises from the compartmentalization of ribosomal DNA (rDNA). In budding yeast, rDNA is localized to chromosome XII, consisting of 150 tandem repeats of ~ 9 kb each. rDNA repeats in higher eukaryotes such as human are more dispersed, with repeats on five different chromosomes, but nonetheless they have similar properties regarding rDNA structure [7]. 

The pericentromere and nucleolus have a number of similarities between their domains. These include condensin/cohesin localization and proteins that regulate the formation of DNA loops. Transfer RNA (tRNA) genes, enriched in condensin and cohesin, are also located within the pericentromere region and tethered to the nucleolus. Replication fork stalling occurs across both centromeres and rDNA repeats, along with a propensity for control of genomic recombination. Lastly, while the nucleolus is a well-identified liquid phase separated region, emerging evidence suggests that the pericentromere may have similar properties. Here, we discuss in detail these commonalities between the pericentromere and the nucleolus.

## 2. Common Features in Both the Pericentromere and the Nucleolus

### 2.1. DNA Loops Are Enriched in the Pericentromere and Nucleolus 

While DNA looping was first observed in 1906 in salamander eggs [8], DNA loops have since been thoroughly documented in a number of different species [9]. These loops can potentially regulate many functions within the cell, including transcription, recombination, and replication [6,10]. Though DNA loops are present in various locations throughout the genome, both the pericentromere and the nucleolus are regions of high loop density that are controlled by unique protein interactors.

**Figure 1:**
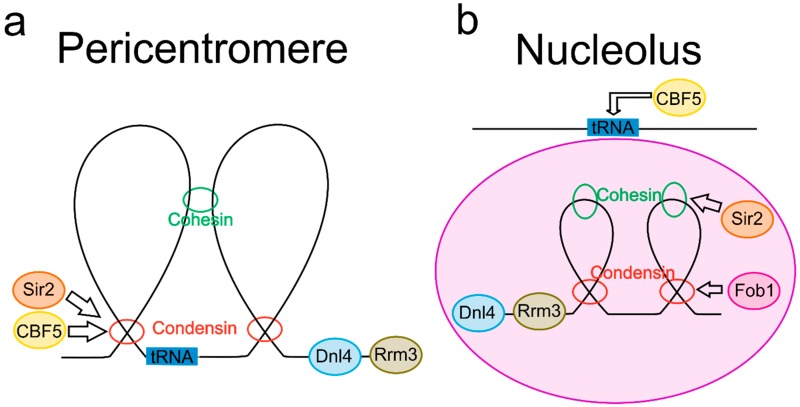
DNA loops in the pericentromere and nucleolus. (**a**) Pericentromere loop schematic. Condensin extrudes DNA in the pericentromere [12,17,18,34] while cohesin radially links nearby loops [11,12]. Pericentric condensin enrichment is controlled by both Cbf5, a small nucleolar ribonucleoprotein, [35] and the histone deacetylase Sir2 [17]. DNA helicase Rrm3 regulates replication fork stalling at the pericentromere [36], and DNA ligase 4 (Dnl4) regulates segregation with a potential role in pericentric fork stalling as well [37,38]. tRNA genes are located in the pericentromere [39,40,41], and are associated with both condensin and cohesin [5,41,42]. (**b**) Nucleolus loop schematic. Condensin [43,44,45,46] and cohesin [23] both regulate loop formation in rDNA. DNA replication fork blocking protein (Fob1) regulates enrichment of condensin in rDNA [47], whereas Sir2 regulates cohesin rDNA localization [48]. Dnl4 [37] and Rrm3 [36] both control fork stalling at rDNA repeats. tRNA genes are tethered to the nucleolus in a Cbf5-dependent manner [49].

In the pericentromere, highly looped DNA has been found in budding yeast (*Saccharomyces cerevisiae*) [11,12] as well as in multicellular organisms such as *Xenopus laevis* [13] and chicken cells [14]. In budding yeast, centromeres from the 16 chromosomes cluster together into a disc approximately 50 nm by 250 nm, which connects to the microtubule plus-ends (for review, see [6]). From here, the pericentric region consists of protruding intramolecular centromere loops, or C-loops, that are formed by loss of sister chromatid cohesion, with radial sub-loops forming off of each C-loop [11,12,15,16] (Figure 1A). The structural maintenance of chromosome (SMC) proteins condensin and cohesin have both been implicated in forming and maintaining these pericentromeric loops in budding yeast [11,12,17,18]. It has been proposed that these loops play an important role in maintaining the mechanics of the pericentromere [15,16], and may provide a mechanism for chromatin condensation [19,20]. Furthermore, the loops generated between the repeat sequences found in higher eukaryotic centromeres may also facilitate recombination [21].

The rDNA present within the nucleolus also features characteristic looping behavior (Figure 1B). In budding yeast, fluorescence in situ hybridization (FISH) staining of the entire rDNA indicates a loop-like structure in nucleoli [22]. Similar to the pericentromere, SMC proteins cohesin and condensin have both been implicated in rDNA loop formation. Cohesin mutations result in reduced looping of the rDNA genes for 35S and 5S in budding yeast [23], which may affect their transcription [24]. Live cell imaging of rDNA in condensin mutants further implicates condensin in the dynamics of loop formation, with time-lapsed imaged mutants displaying a delay compared to wild-type in loop formation [25]. Condensin-mediated loop extrusion of rDNA is further supported by globally generated Hi-C contact maps [26]. Both nucleolar transcription factor 1 (UBF), a mammalian protein containing high mobility group (HMG) dox domains, and its yeast homolog, high mobility protein 1 (Hmo1), bind preferentially to actively transcribed rDNA genes [27,28] and are enriched in the nucleolus [29]. Electron spectroscopic imaging in *Xenopus* suggests that UBF dimers bend approximately 150 bp rDNA into a loop formation [30,31]. While Hmo1 has yet to specifically be identified in the looping feature of rDNA, the reliance on the HMG box for the looping activity of UBF suggests that Hmo1 may have a similar role [32]. Also in *Xenopus*, immunostaining indicates that Pol III sites (which bind to nucleolar tRNA genes) are localized to DNA loops [33], suggesting an additional nucleolar DNA looping site.

### 2.2. SMC Proteins in the Pericentromere and Nucleolus Display Common DNA Regulatory Roles

Structural maintenance of chromosome (SMC) protein complexes, such as condensin and cohesin, are essential to regulation of chromosome function and structure. Condensin and cohesin are enriched both in the pericentromere [3,42,50,51] and in the nucleolus [52,53] in a variety of different organisms, suggesting an important conservation of function. These proteins play an important role in cohesion between sister chromatids, chromosome segregation, DNA replication, DNA damage repair, and DNA loop formation. In eukaryotes, SMC proteins form heterodimers, creating a V-shaped molecule with a variable conformation [54]. While there are two different identified condensin complexes (condensin I and II), only condensin I has been found in fungi such as budding and fission yeast [20].

The SMC heterodimer in condensin I/II consists of Smc2/Smc4. Non-SMC subunits in condensin I includes chromosome associated protein H (CAP-H; a member of the kleisin protein family), and HEAT repeat (which consists of Huntingtin elongation factor 3 [EF3], protein phosphatase 2A [PP2A], and the yeast kinase, target of rapamycin 1 [TOR1])-containing chromosome associated protein D2 (CAP-D2) and chromosome associated protein G (CAP-G), whereas condensin II contains CAP-H2, CAP-D3, and CAP-G2 [20]. As an SMC protein complex, condensin has a number of different roles in regard to DNA regulation. As its name suggest, condensin is essential for chromosome condensation, or heterochromatin formation [52,55]. More recently, studies have found that condensin is involved in extruding pericentric DNA loops [12,17,18] as well as cross-linking DNA in trans [56]. Live imaging of yeast condensin along double-tethered λ -DNA also showed that condensin mediates loop extrusion [34]. The localization of condensin is further regulated by proteins such as the histone deacetylase Sir2. Sir2 contributes to the axial position of condensin in the pericentromere, in which condensin is located proximal to the yeast mitotic spindle. In yeast lacking Sir2, condensin becomes more radially displaced, distal to the spindle and perpendicular to the spindle axis, and can appear as a bilobed distribution similar to pericentric cohesin [17]. Condensin also has a role in positive supercoiling of DNA in both *Xenopus* [57] and budding yeast [58], which may promote proper segregation as positively supercoiled DNA is more resistant to pulling forces [6,59]. Meanwhile, in rDNA, FISH studies in budding yeast indicate that condensin is necessary for rDNA looping [43,44,45,46]. DNA replication fork blocking protein Fob1, a known rDNA binding protein, is responsible for loading condensin onto rDNA repeats in yeast [47].

Cohesin contains a Smc1/Smc3 heterodimer, as well as two other subunits: Scc1 (also referred to as Mcd1 or Rad21) and Scc3 (also known as SA) [54]. Cohesin can form a ring-like molecule [60,61] among many other possible configurations [62,63,64]. While first identified for its prominent role in sister chromatid cohesion [65], the exact mechanism of the cohesion ability of cohesin remains debated. Possible models include the ring model, in which a single cohesin molecule embraces both sister chromatids, the handcuff model, in which two cohesin rings on either sister chromatid bind together, and the bracelet model, in which a cohesin oligomer wraps around the sister chromatids [66]. Chromatin immunoprecipitation (ChIP) assays on yeast strains with mutated alleles of cohesin subunit Mcd1 also revealed that cohesin preferentially binds the pericentromere versus the chromosome arms [67]. In vivo studies using yeast indicate that during metaphase, cohesin is radially displaced from the pericentric DNA [11,12], which is dependent on its ability to passively diffuse along the chromosome [18], and suggests that it plays a role in linking the C-loops generated by condensin. However, other studies suggest a cohesin-mediated loop extrusion model, particularly during interphase [62,68]. In mammalian cells, ChIP-seq in combination with Hi-C indicates that cohesin is localized to topological associated domains (TADs), an indicator of loop formations [69], and further studies suggest cohesin may directly regulate these loops [70,71,72,73]. Outside of the pericentromere, cohesin is recruited to both tRNA genes and rDNA sites in a Sir2-dependent mechanism [48,74]. Cohesin is further involved in the cohesion of rDNA sister chromatids in budding yeast [65,75], and controls mitotic rDNA organization [76]. Mutations of cohesin are associated with both disorganization of the nucleolus and reduced looping of rDNA [23], concurrent with less rRNA production and subsequent protein translation [77], suggesting a particular role for cohesin at this locus.

Condensin and cohesin feature small ubiquitin-like modifier (SUMO) sites, or sumoylation sites [78], a reversible modification which affects their distribution in both the pericentromere and at rDNA sites. Deletion of Ulp2 (also referred to as Smt4), an isopeptidase that removes SUMO from proteins, causes a decrease in pericentric condensin clustering [79], as well as a decrease in condensin localization to rDNA sites [80] in budding yeast. This result is consistent with decrease in tension at the pericentromere [79] and lack of sister chromatid cohesion [81], suggesting a role for sumoylation in these activities of pericentric condensin.

DNA topoisomerase II (Top2) has distinct interactions with condensin and cohesin in both the pericentromere and rDNA in a variety of eukaryotic species [82,83,84]. In the pericentromere, Top2 has an essential role in regulating mitotic chromosome structure and tension [81,85]. Depletion of condensin in *Drosophila* disrupts Top2 centromeric localization [86], suggesting that condensin plays a role in regulating Top2 localization. A similar dependence for condensin-mediated Top2 is observed at the rDNA locus in budding yeast, with both reduced binding of Top2 in the absence of condensin as well as a lack of restoration of segregation defects with Top2 overexpression in the condensin mutants [83]. Sumoylation sites are present in Top2 [81], potentially influencing its activity. Deletion of either Top2 or isopeptidase Ulp2/Smt4 in budding yeast causes a decrease in compaction at the pericentromere during metaphase [79] as well as increased pericentromere stretching [85]. These data suggest that Top2 activity, coordinated by sumoylation, regulates pericentromere dynamics. In yeast lacking cohesin, in which biorientation of sister kinetochores is lost during mitosis, depletion of Top2 actually restores biorientation, indicating that linkage between sister chromatids is a balance between cohesion and catenation [87]. Similar findings have also been observed in DT40 chicken cells [88]. A combination of ChIP-seq and Hi-C analysis indicates that human Top2 associates with cohesin subunits and rDNA-binding proteins [84], confirming a potential role of Top2 at these locations.

In mammalian cells, a zinc finger protein called CCCTC-binding factor (CTCF) is involved in forming chromatin loops [89,90] and associates with cohesin [91,92]. CTCF can function as an insulator, preventing interaction between active and inactive chromatin and blocking enhancer activity [93]. While CTCF has not been identified in lower eukaryotic species such as *Saccharomyces cerevisiae*, *Schizosaccharomyces pombe*, and *Caenorhabditis elegans* [94], transformation studies suggest that CTCF has a similar insulating function in yeast [95]. Furthermore, members of the Ctf19/COMA complex, which regulates pericentromeric cohesin enrichment [51,96,97], play a vital role pericentromere loop formation [98], perhaps similar to mechanism by which CTCF mediates cohesin at the base of loops [99]. This suggests that the Ctf19/COMA complex could function as the yeast equivalent of CTCF. ChIP-seq analysis also indicates that CTCF associates with Pol III sites (tRNA genes) [100], which as discussed below, are tethered to the nucleolus and localized in the pericentromere. CTCF further regulates rDNA in human cells [101,102], and localizes to the nucleolus in mammalian cells and *Drosophila* [103,104]. Knockdown of condensin increases CTCF binding to rDNA, suggesting a role for condensin in negatively regulating CTCF [105]. These data therefore suggest a common role for CTCF, possibly via association with SMC proteins, in regulating both pericentric and nucleolar DNA.

### 2.3. tRNA Genes Are Localized to Both the Pericentromere and the Nucleolus

Transfer ribonucleic acid (tRNA) genes, also referred to as tDNA, are short sequences located throughout the genome and are bound by transcription factor RNA polymerase III (Pol III) [106]. These genes, of which there are 274 in yeast and approximately 450 in humans, are dispersed throughout the genome. However, nucleotide sequencing in fission yeast has shown that some tRNA genes are localized in the pericentromere in fission yeast [107], and FISH studies have identified pericentric tRNA genes in both fission and budding yeast [39,40,41]. Tfc1, a subunit of the Pol III transcription factor complex, was also found to be localized in the pericentric region in budding yeast [35]. Some tRNA genes are also located in the periphery of the nucleolus in fission and budding yeast [40,41,108], and early processing of tRNAs has previously been found to occur in the nucleolus in yeast [109]. It has further been shown that the tethering of tRNA genes to the nucleolus is influenced by proximity to centromeres, with closer proximity of Pol III-transcribed genes to centromeres associated with less nucleoli association [110]. This suggests an additional level of regulation of tRNA gene localization that is influenced by Rabl configuration-like organization, consistent with chromosome arms extending away from centromeres towards the nucleolus [111,112].

tRNA genes are sites of enriched cohesin [42] and condensin [5,41]. Interestingly, condensin mutations result in loss of nucleolar clustering of tRNA genes, suggesting that condensin plays a role in tRNA localization [41]. Deletion of tRNA genes on chromosome III in budding yeast disrupts not only condensin localization to tDNA sites, but also affects centromere–centromere interaction [112], suggesting effects of tRNA genes on chromosome structure. Cohesin regulates tRNA activity, with mutations that cause human cohesinopathies resulting in defects in tRNA gene-mediated silencing when expressed in budding yeast [113]. The histone deacetylase Sir2, which as previously mentioned affects axial condensin localization at the pericentromere [17], is similarly responsible for enrichment of condensin and cohesin at tRNA sites [74].

Cbf5, a small nucleolar ribonucleoprotein, further regulates tRNA distribution. Mutations in Cbf5 disrupts both the nucleolar localization of pre-tRNAs as well as alleviating tRNA gene-mediated silencing [49]. Cbf5 also regulates pericentric condensin; in budding yeast, Cbf5 mutants have decreased condensin enrichment at the pericentromere [35], suggesting that condensin regulation may underlie the effects of Cbf5 on nucleolar tRNA localization.

Certain DNA elements called chromatin barriers or insulators, which are present in multiple eukaryotic species, play a role in structurally defining functionally distinct chromatin regions [114]. In particular, tRNA genes have been shown to separate heterochromeric DNA from unsilenced regions. In fission yeast, a centromeric tRNA gene was found to play a role in defining centromeric heterochromatin and normal meiotic segregation [115], and is dependent on Pol III activity [116]. In human cells, tRNA genes have a similar function, with multimerized tDNAs increasing enhancer blocking [117]. Similarly, tRNA-mediated gene silencing is dependent on its nucleolar localization in budding yeast [40]. In addition, tRNA genes represent sites of replication fork pausing in budding and fission yeast, possibly due to their high rates of transcription [118,119]. Furthermore, in both specific tRNA genes and centromeres, there is a dependency on replisome progression complex member Tof1, but not Mrc1, for replication fork pausing [120]. The function of tRNA as a chromatin barrier in multiple eukaryotic species suggests a distinct conservation of this mechanism.

### 2.4. Replication Fork Stalling in Pericentromere and rDNA

Both centromeres and rDNA repeats in the nucleolus are characterized by blockades that disrupt fork progression. While highly repetitive DNA sequences are thought to promote replication fork pausing [121,122,123], even the ~125 bp non-repetitive point centromeres in yeast feature fork stalling [120,124]. A complex of S-phase checkpoint proteins (Tof1, Mrc1, Csm3) that are responsible for slowing DNA synthesis in the presence of DNA damage localize specifically to replication forks in budding yeast [125]. Tof1 in particular is required for fork pausing at yeast centromeres [120]. Deletion of Csm3, which interacts directly with Tof1 [126], is important for establishment of fork pausing [127]. DNA helicase Rrm3 promotes replication fork progression at multiple genomic sites, including tRNA genes, rDNA, and centromeres [36,128]. Csm3 may facilitate this fork pausing by blocking Rrm3 helicase-induced progression through replication forks [129], suggesting a common mechanism of stalled forks in both the nucleolus and the pericentromere.

Chl4, Iml3, and Mcm21, proteins that are members of the Ctf19 complex in yeast (analogous to the constitutive centromere associated network, or CCAN, in mammals), are involved in kinetochore assembly at the centromere [130]. Interestingly, Chl4, Iml3, and Mcm21 are all required for pericentric cohesin enrichment [96,97]. Loss of either Iml3 or Chl4 causes a decrease of pericentric cohesin, which is counteracted by slowing replication with hydroxyurea treatment [96]. The cohesin-loading function of these proteins could therefore be instrumental to ensuring that pericentric cohesin is in place prior to the replication fork, ensuring proper cohesion of the resulting sister chromatids. A yeast model using a conditional dicentric strain, which allows the study of de novo kinetochore assembly, demonstrated that Chl4/Iml3/Mcm21 mutants all suppress dicentric breakage [131]. The lack of breakage that is normally induced in a dicentric strain is consistent with a lack of de novo kinetochore assembly in these mutants. Furthermore, de novo kinetochore assembly in Chl4/Iml3 mutants is rescued by pausing replication using hydroxyurea [98]. By slowing the replication process, this may allow additional time for these mutants to resume proper kinetochore assembly.

At the rDNA locus, stalling at replication forks has been well-established in both yeast and mammals [132]. In a single 9 kb rDNA repeat in budding yeast, the 35S gene is transcribed by Pol I, followed by the 5S gene that is transcribed by Pol III in the opposing direction. A replication fork barrier (RFB) is located at the 3’ end of the 35S gene, allowing replication to occur through 35S but blocking replication in the opposing direction [133]. At the human rDNA locus, however, the replication fork barrier functions in a uniquely bi-directional manner, blocking replication from occurring in both directions at this junction [134]. In yeast, a protein called Fob1 is required for RFB activity at the rDNA locus [135,136]. Fob1 co-localizes with rDNA-binding protein Hmo1 [29] and also condensin [47], proteins that as discussed previously may be involved in DNA looping. Similarly, atomic force microscopy imaging indicates that the RFB sequence may actually wrap around Fob1 in a nucleosome-like fashion [136].

Top2, which as previously mentioned associates with SMC proteins, plays an important role regarding replication termination both in the pericentromere as well as in rDNA repeats. Termination regions (TERs) are located at the point of two converging replication forks, contain fork pausing elements [137], and are crucial for terminating replication [138]. In budding yeast, TERs are particularly concentrated near centromeres, and mutation of Top2 causes double-stranded breaks and recombination at these sites [138]. Top2 mutants result in repair checkpoint activation that is counteracted by a Top2-Fob1 double mutant, implying that Top2 mediates proper replication termination in rDNA as well [139].

DNA ligase 4 (Dnl4), a protein involved in non-homologous end-joining DNA repair [140], may play a role at the replication fork barrier in rDNA sites. Dnl4 interacts with replication fork-associated Sgs1 to prevent fork breakage-mediated events in rDNA [37]. While deletions of Dnl4 in budding yeast may influence segregation events at the pericentromere [38], it remains unclear if Dnl4 may have a similar role specifically in pericentric fork pausing.

### 2.5. Recombination Control in the Pericentromere and rDNA

The similarities between the pericentric region and nucleolar rDNA suggests that these two regions may also have similar features in regard to control of recombination and resulting genomic instability. Highly repetitive regions of the genome, such as those in regional centromeres of mammals and rDNA in eukaryotes, are susceptible to mitotic recombination. While recombination during meiosis tends be greatly repressed near the centromere in a variety of species [141], in mitosis, recombination occurs at budding yeast pericentromeres in the form of gene conversion [142], though this may occur at reduced levels closer to the centromere [143]. In mammals, however, which feature regional centromeres with high numbers of repeat sequences, mitotic recombination is a common occurrence. Using chromosome-oriented FISH to specifically target centromere repeats in mouse cells, it has been shown that there are extremely high mitotic recombination events at centromeres compared to the rest of the genome [144]. As mentioned previously, it has also been proposed that the highly repetitive sequences found in higher eukaryotes promotes recombination, which further drives the loop formation that is necessary for proper centromere function [21].

Replication fork stalling further facilitates recombination events [145]. Double-stranded breaks sometimes occur as a result of stalled replication forks [146,147], which are commonly repaired by either homologous recombination in yeast, or non-homologous end-joining in mammals. The rDNA-binding protein Fob1, which is essential for RFB formation, is particularly important for facilitating these recombination events [148,149]. As demonstrated in mutant yeast strains with lower copy numbers of rDNA, this activity of Fob1 is also dependent on the rate of transcription, with higher transcription correlating to more recombination even in Fob1 mutants [150]. Recombination hot-spot (HOT1), a DNA element that increases levels of inter- and intrachromosomal homologous recombination between repeats [151], is highly prevalent surrounding rDNA repeats. Analysis of HOT1 mutants indicated that only the set containing mutated Fob1 had defects in homologous recombination, suggesting that Fob1 is an important mediator at these locations [135]. Kobayashi et al. further demonstrated that Fob1 mediates the expansion/contraction of rDNA repeats [149]. Whether fork stalling might influence recombination at the pericentric region, however, has yet to be determined.

Sir2, which as previously mentioned affects localization of condensin/cohesin at the pericentromere and rDNA sites, appears to negatively influence recombination in rDNA repeats. Sir2 mutants have a reliance on recombination genes (Rad50 and Rad52) that are dispensable in wild-type strains [152], suggesting a unique recombination pathway controlled by Sir2. Deletion of Sir2 also increases the number of rDNA repeats, and decreases rDNA-associated cohesin [48], suggesting that Sir2 may mediate or regulate levels of recombination by enhancing sister chromatid cohesion. Interestingly, in the pericentromere of fission yeast, cohesin prevents double-stranded breaks and the resulting recombination events from occurring during meiosis [153]. These data suggest a potential role for Sir2 and cohesin in controlling recombination events in both the pericentromere and rDNA.

Top2, as a mediator of replication fork progression and sister chromatid decatenation, may regulate recombination events at the pericentromere and at rDNA sites. Top2 mutants have enhanced recombination events at TERs, regions that are concentrated near centromeres [138], and enhanced recombination is observed at rDNA sites in Top2 mutants [154], suggesting that Top2 may suppress recombination at these regions. Conversely, the association between Top2 and cohesin/CTCF [84] at potential loop anchor points may actually promote rearrangement events. In mammalian cells, double-stranded breaks induced by the anti-cancer drug etoposide corresponds with sites occupied by CTCF and Top2 [155,156]. Meanwhile, double-stranded breaks are decreased in Top2 mutants [155]. These data suggest that Top2 may have unique effects on genomic stability, particularly through its interactions with proteins such as cohesin.

### 2.6. Phase Separation in the Nucleolus and Pericentromere

Phase separation as a mechanism for defining discrete departments within the cell has been a rapidly growing field within cell biology. The nucleolus, which consists of liquid–liquid separated phases, also known as biomolecular condensates [157], has been the target of many such studies. Liquid–liquid phase separation (LLPS) has been identified in the nucleolus of *Xenopus* [158,159] as well as *C. elegans* [160]. Photobleaching experiments in budding yeast further suggest an organized network of the nucleolus, with distinct segregation of nucleolar proteins following mitosis [161]. Membraneless organelles of not only the nucleolus but also those such as stress granules and nuclear speckles are characterized by RNA-protein interactions [162], suggesting that these interactions may promote phase separation. Supporting this notion, mutation of the RNA recognition motif of nucleolar protein NPM1 prevents the formation of liquid-like droplets [159,163]. LLPS is thought to be crucial in defining the organization of nucleoli [159], facilitating the role of nucleolar sub compartmentalization in RNA processing and ribosome biogenesis [164]. In human cell lines, the liquid-like state of the nucleolus is further involved in quality control of misfolded proteins [165].

In addition to LLPS, polymer–polymer phase separation (PPPS) may also play a role in compartmentalizing not only the nucleolus but also in defining chromosome territories. Polymer models suggest that entropic forces generated by chromatin polymers constrain chromosome territories [166,167,168,169]. Furthermore, computational modeling of nucleolar structure suggests nucleolar phase separation may be driven by polymer crowding, even without assuming the presence DNA binding factors [170]. Bead-spring models of chromatin dynamics further suggest that nucleolar PPPS is formed by chromosomal cross-linking and DNA loop formation [171]. In addition, this notion that DNA loops drive PPPS in the nucleolus suggests that a similar mechanism may regulate PPPS at the pericentromere, which as previously discussed is characterized by the formation of DNA loops.

While the pericentromere is less well-studied compared to the nucleolus in regard to potential LLPS properties, there is emerging evidence that supports such phase separation. The chromosomal passenger complex (CPC), which includes the kinase Aurora B as well as other subunits such as INCENP and borealin, regulates tension between sister kinetochores, ensuring proper segregation of sister chromatids [130,172]. *In vitro* experiments indicate that the non-kinase subunits of the CPC form liquid-like droplets at physiological centromere concentrations, and experiments using HeLa cells further suggest that phase separation induced by CPC component borealin mediates its location to the inner centromere [173]. The authors found that alpha satellite RNA was associated with the liquid-like droplets, suggesting a potential role of CEN RNA in formation of this liquid–liquid phase, similar to nucleolar RNA/protein interactions facilitating LLPS [162]. Interestingly, Aurora B mediates pericentric enrichment of condensin in HeLa cells [174], suggesting a potential interplay for other pericentric proteins in mediating phase separation. Furthermore, interphasic centromeres can localize to the nucleolus in both *Drosophila* and human cells [175,176,177]. In human cells, the CPC component INCENP associates with centromeric alpha satellite RNA, targeting the interphasic nucleolar localization of centromeres [178]. Whether this activity may be linked to a potential role of CPC components in liquid–liquid phase separation has yet to be determined, however.

Repetitive heterochromatin, such as the alpha satellite repeats present in regional centromeres, has been shown to promote liquid–liquid phase separation. In particular, heterochromatin protein 1α (HP1α), a protein localized to heterochromatin in mitotic centromeres in higher eukaryotes [179], is responsible for liquid-like droplet formation in *Drosophila* and mammalian cells [180,181]. While HP1 has not been identified in budding yeast, the histone deacetylase Sir2 may have a similar role in regulating heterochromatin [182]. It has further been suggested that phase separation of these repetitive DNA sequences may drive chromatin organization and folding [183], suggesting a potential mechanism at centromeres and in the nucleolus.

## 3. Conclusions

As two seemingly discrete regions, the pericentromere and nucleolus have a number of commonalities in regard to their features and regulatory mechanisms. The similar chromatin structure of both the pericentromere and rDNA, regulated in part by SMC proteins, may impart similar features such as fork pausing, tRNA tethering, regulation of genomic instability, and phase separation. Mechanisms underlying such activities in one region may therefore be used to guide studies of similar activities in the other region.
